# Introducing an enhanced cadre of pharmacy assistants to improve dispensing, management, and availability of medicines at the health centre level in Malawi

**DOI:** 10.1186/2052-3211-7-S1-O23

**Published:** 2014-12-17

**Authors:** Matthew Ziba, Joseph Babigumira, Jessica Crawford, John Kandaya, Charles Chimenya, Alisa Jenny, Solomon Lubinga, Charles Matemba, Erin Larsen-Cooper, Andy Stergachis

**Affiliations:** 1 VillageReach, Seattle, WA, USA; 2 Malawi College of Health Sciences, Blantyre, Malawi; 3 Ministry of Health, Lilongwe, Malawi; 4 University of Washington, Global Medicines Program, Seattle, WA, USA

## Background

VillageReach, in partnership with the Malawi Ministry of Health, the Malawi College of Health Sciences and the University of Washington Global Medicines Program, is addressing key barriers to medicines availability by implementing a new approach to training, deployment, and support of an enhanced pharmacy assistant cadre. Key aspects of the program include curriculum redesign to include more content to enhance skills in supply chain management and an extensive practicum component at public health facilities.

## Method

Student enrolment and examinations are monitored by the college. A baseline assessment and monthly data collection are conducted at health facilities prior to and during student practicum placements. Information on stock-outs, reporting timeliness and accuracy, dispensing quality, and pharmacy and storeroom conditions are collected during supervision visits. A population-based survey examining access to medicines at the community level was conducted at baseline and will be repeated annually as part of an impact evaluation.

## Results

All 50 students from the first cohort successfully completed their first year of coursework and practicum and 100 new students enrolled in 2014. District hospitals that hosted students experienced improved pharmacy and storeroom conditions, increased on-time reporting, and improved dispensing standards. By the time of this conference, six months of data will be available from practicum health centres including; stock-out rates, changes in storeroom conditions, storeroom management guidelines and amount of clinical staff time spent on logistics tasks. Baseline data on community access to and use of medicines from the population-based survey will also be available for presentation.

## Discussion

The training program is designed such that students rotate through practical settings after 10 weeks of in-class coursework. This allows for more skills-based training and for more immediate improvements at the health facilities. With 100% student retention coupled with improved supply chain performance at practical training sites, the Pharmacy Assistant Training Program is a promising solution for countries with limited health workforce and supply chain challenges. We expect even greater improvements at health centres over time where students will have more direct control over supply chain management for public health facilities.

## Lessons learned

The program is showing promise that skills-based training of pharmacy certificate students improves the performance of medicines supply chain and increases access to medicines in public health facilities.

